# Case Report : Giant phyllodes sarcoma: Case Report of total mastectomy with immediate latissimus dorsi flap reconstruction – A surgical and aesthetic challenge

**DOI:** 10.12688/f1000research.159059.1

**Published:** 2025-01-27

**Authors:** Dhekra Toumi, Chayma Cheikh Mohamed, Ahmed Hajji, Imen Ghaddab, Malak Medemagh, Hanane Lazreg, Yasmine Ben Ali, Sana Alouani, Jawaher Hammedi, Selma Souilah, Rihab Barouni, Hiba Ben Abdelhafidh, Sana bouakez, Thana Mahfoudhi, Olfa Zoukar, Haifa Bergaoui, Raja Faleh

**Affiliations:** 1 Obstetrics and Gynecology Department, Faculty of Medicine, Monastir Maternity and Neonatology Centre, University of Monastir, Monastir, Monastir, 5030, Tunisia

**Keywords:** Phyllodes sarcoma, Giant breast tumor, mastectomy, Latissimus dorsi flap reconstruction, Breast oncology, Reconstructive surgery, body image, quality of life

## Abstract

Phyllodes tumor of the breast is a rare fibroepithelial tumor, constituting less than 1% of all breast neoplasms. These tumors are characterized by rapid stromal growth and can reach significant sizes, particularly in malignant cases, necessitating aggressive surgical management due to their high recurrence risk. In this case report, we present a 40-year-old Tunisian woman diagnosed with a giant phyllodes sarcoma. The patient experienced considerable physical and psychological impacts, including impaired mobility, body image disruption, and professional limitations. Clinical examination and imaging confirmed the presence of a large lobulated mass in the left breast, with no signs of distant metastasis. A multidisciplinary team recommended a total mastectomy with immediate reconstruction using a latissimus dorsi myocutaneous flap. This approach aimed to restore breast contour while addressing both oncologic and aesthetic needs. The procedure was successful, with the excised tumor weighing 7 kg. Postoperatively, the patient received close monitoring and psychological support, emphasizing the importance of a holistic approach to recovery. This case underscores the complexities of managing giant phyllodes sarcoma, highlighting the value of a timely surgical intervention combined with effective reconstructive techniques. It also emphasizes the need for individualized treatment plans that consider both functional and aesthetic outcomes to optimize patient recovery and quality of life

## Introduction

Phyllodes sarcoma of the breast is a rare tumor, accounting for less than 1% of all breast neoplasms. ^1, 2^ Characterized by rapid stromal growth, these tumors can reach considerable sizes, particularly in malignant forms, which often necessitate aggressive surgical intervention due to their high recurrence potential. ^3, 4^ Although typically non-metastatic, giant phyllodes tumors pose unique challenges because of their significant size and the accompanying functional and aesthetic impacts. ^5^ Managing large phyllodes sarcomas generally requires a total mastectomy, often paired with immediate breast reconstruction to minimize the physical and psychological consequences for the patient. ^6, 7^ The latissimus dorsi myocutaneous flap is a commonly employed technique in such cases, allowing for restoration of the breast shape using a segment of muscle, fat, and skin from the back. ^8– 11^ Despite its frequent use, this procedure involves specific complexities related to tissue viability and achieving satisfactory aesthetic outcomes, particularly with exceptionally large tumors. ^12, 13^

In this report, we present the case of a patient with a 7 kg giant phyllodes sarcoma, treated with a total mastectomy followed by immediate reconstruction using a latissimus dorsi flap. We explore the clinical features, therapeutic strategies, and both the functional and aesthetic results of this approach, contributing to the literature on breast oncology and reconstructive surgery.

## Case presentation

We present the case of a 40-year-old Tunisian woman with no family history of breast cancer who had previously undergone a thyroidectomy for a benign nodule. The patient, a seamstress, reported a rapidly growing left breast mass over five months, leading to significant deformity and asymmetry, severely affecting her quality of life.

Physically, the patient experienced persistent chest heaviness and pressure, limiting her ability to perform daily activities such as bending, standing, or sleeping comfortably. The tumor’s size and weight placed substantial strain on the skin, which became stretched, thinned, and erythematous, with superficial ulcerations due to skin distension and friction from clothing, causing localized pain and increasing the risk of infection. Additionally, she reported chronic thoracic pain, described as a pulling or intense pressure, worsened by movement.

Psychologically, the unchecked tumor growth caused severe body image disruption, leading to a decline in self-esteem and social withdrawal due to the physical burden and the anxiety surrounding the tumor’s uncertain nature and potential malignancy. The tumor also impacted her professional life, as the weight hindered her ability to perform essential tasks, such as bending, lifting her arms, and sitting for long periods, resulting in a loss of productivity and income.

**Figure 1.**
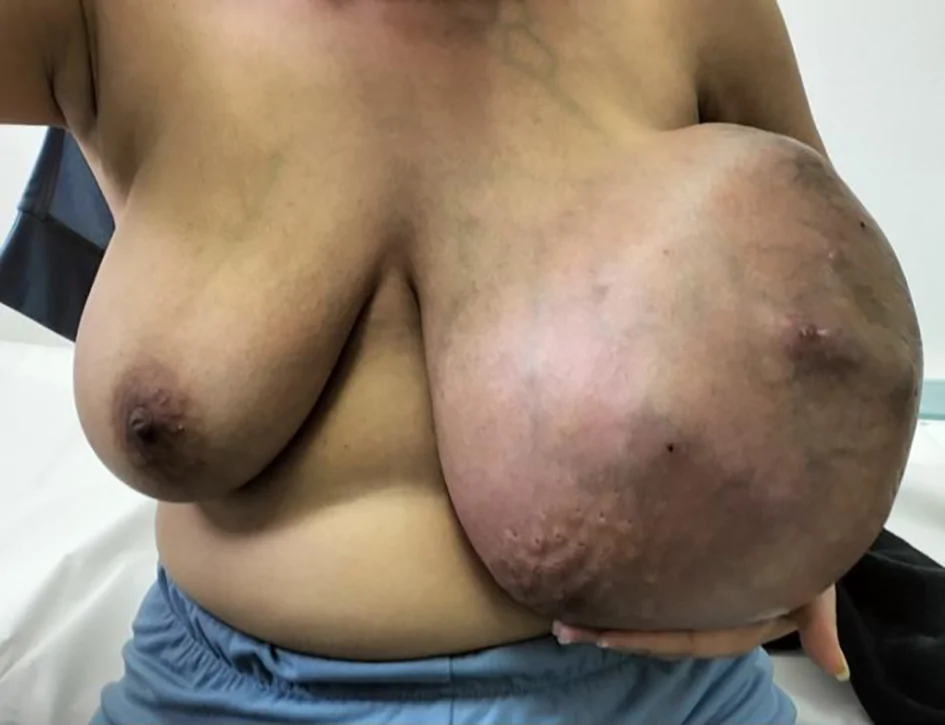
Giant Phyllodes Tumor on Clinical Examination: A 40-year-old female patient presenting presents with a giant phyllodes tumor (30 × 40 cm) in the left breast, accompanied by dilated subcutaneous vessels.

Clinical examination, the tumor was well-defined, firm, and mobile, with an irregular surface and lobulated edges. The skin was erythematous and stretched, exhibiting ulcerations and an “orange peel” appearance. No axillary lymphadenopathy was detected, and there were no signs of distant metastasis ( Figure 1).

**Figure 2.**
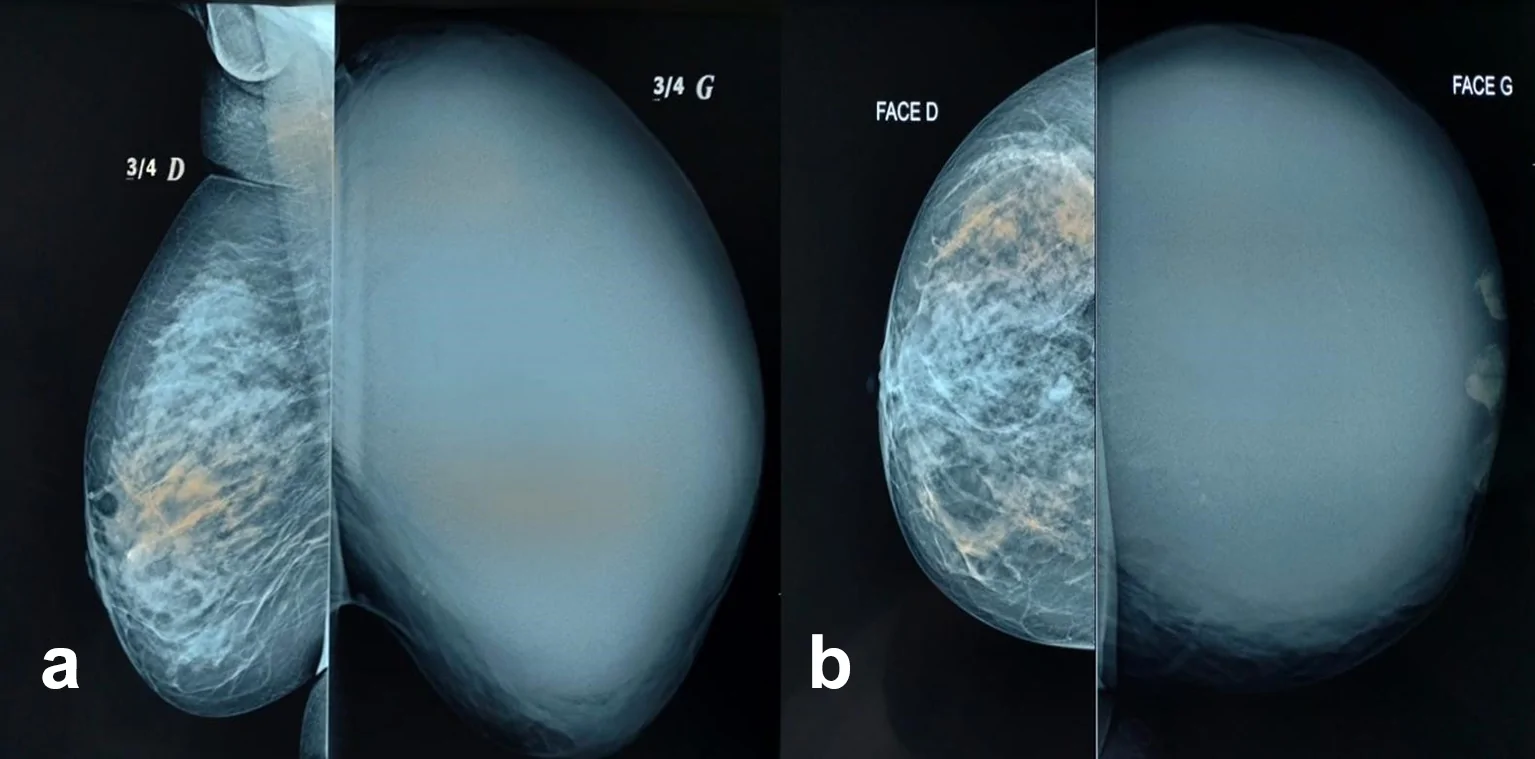
initial mammogram: (a) Oblique view, (b) Frontal view. The initial mammogram revealed a large lobulated mass in the left breast, indicating an abnormal, rounded shape with irregular contours. This mass was associated with architectural distortion.

An initial breast ultrasound suggested the presence of proliferative mastitis in the left breast, complicated by superinfection, alongside fibrocystic changes in the right breast. A bilateral mammogram revealed a 40 × 30 cm lobulated mass in the left breast, associated with architectural distortion and asymmetry. Subsequent thoraco-abdominopelvic CT imaging identified a large tumor in the left breast, along with ipsilateral axillary lymphadenopathy, but no suspicious lesions were observed below the diaphragm. A breast biopsy was performed, confirming the histopathological diagnosis of a phyllodes sarcoma ( Figure 2).

A multidisciplinary team meeting recommended a total mastectomy with 1 cm margins, given the tumor’s size and rapid growth, followed by immediate reconstruction using a latissimus dorsi flap to address the expected aesthetic and psychological impact.

**Figure 3.**
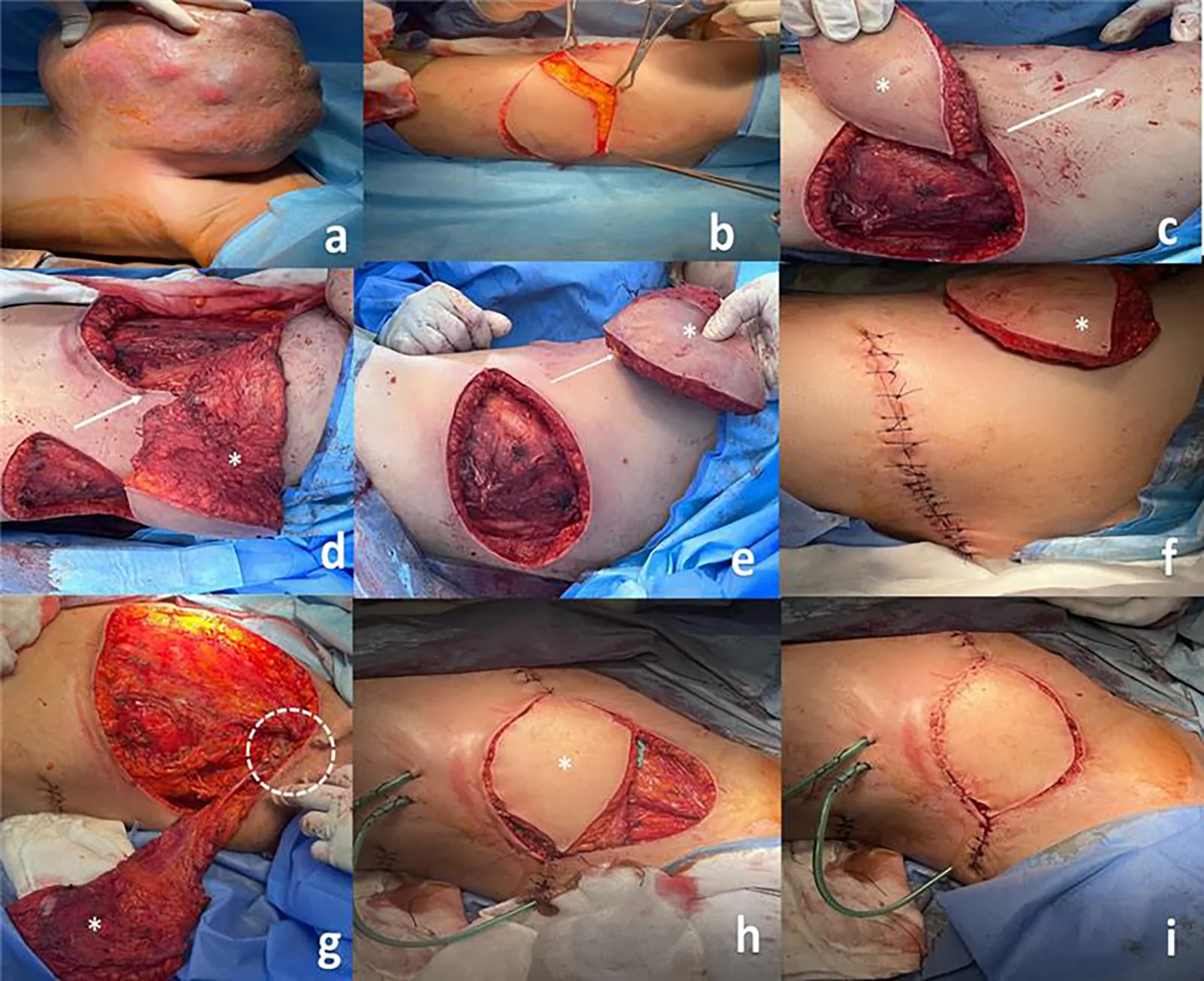
Comprehensive procedure for latissimus dorsi myocutaneous flap harvest and transfer for breast reconstruction. (a) Comprehensive preoperative evaluation (b) Dorsal incision made to harvest the latissimus dorsi myocutaneous flap (c) Creation of a subcutaneous tunnel from the dorsal region to the chest to allow flap transfer: Careful dissection and tissue detachment to create a subcutaneous pocket, while ensuring the preservation of the flap's vascular supply. The white arrow in the image indicates the direction of the tissue dissection and the subcutaneous tunnel created to allow for the flap's passage. (d), (e) Transfer of the flap through the subcutaneous tunnel to the mastectomy bed. (f ) The donor site incision is then meticulously sutured. (g) Careful preservation of key blood vessels during flap harvest, followed by precise flap positioning on the mastectomy bed. The dashed circle in the figure indicates the preserved vascularization, while the asterisk marks the flap. (h) Shaping of the flap to restore breast volume and contour. (i) Attachment of the flap to ensure proper reconstruction.

The procedure began with a thorough preoperative evaluation to assess the required tissue volume and ensure the viability of both donor and recipient sites. Under general anesthesia, the mastectomy was first performed, followed by an incision in the dorsal region to harvest the latissimus dorsi myocutaneous flap, carefully preserving key blood vessels. The flap was then meticulously transferred through a subcutaneous tunnel to the chest, where it was shaped and attached to restore breast volume and contour. Special attention was given to achieving aesthetic symmetry and an optimal cosmetic outcome ( Figure 3).

**Figure 4.**
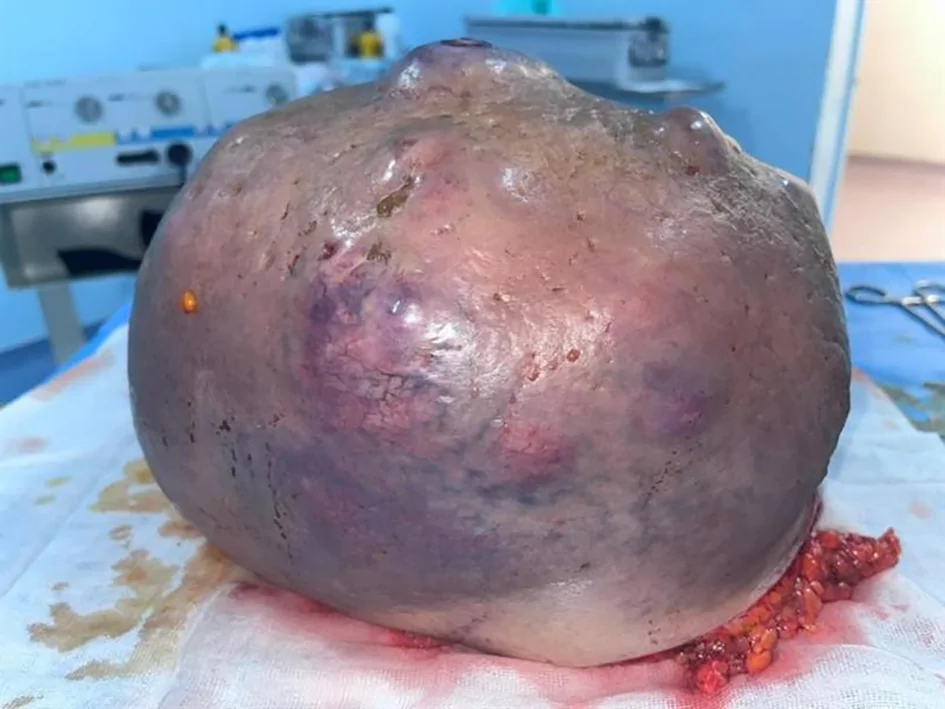
Surgical specimen, tumor weighing 7 kg.

The excised tumor weighed 7 kg, and histopathological analysis reaffirmed the preoperative diagnosis of a high-grade phyllodes sarcoma ( Figure 4).

**Figure 5.**
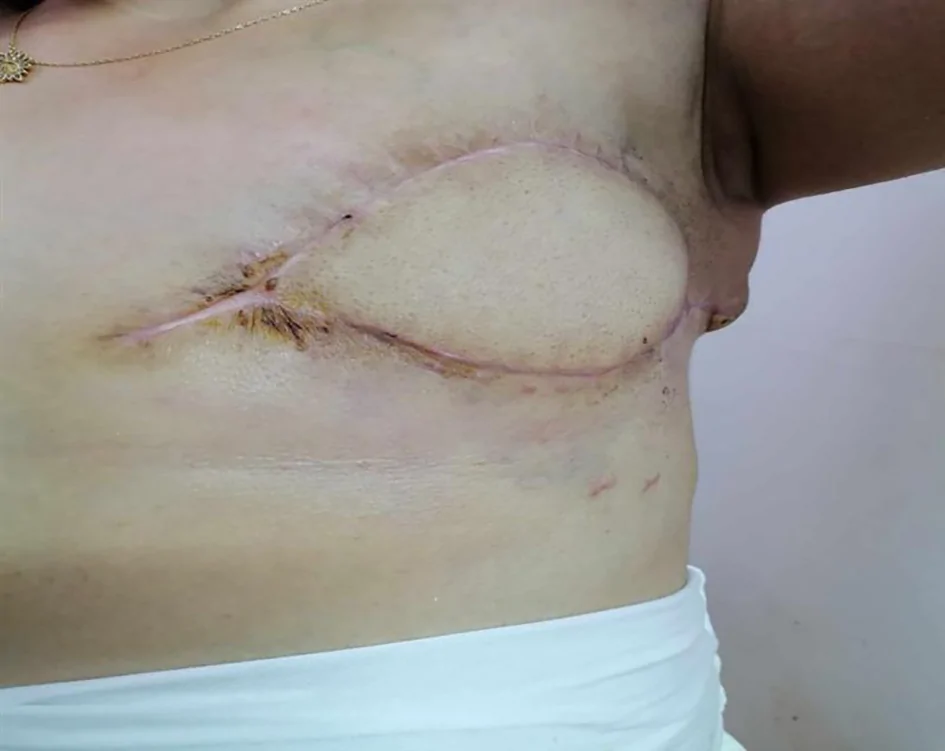
Appearance of breast reconstruction site three months post-surgery.

Postoperatively, the patient was closely monitored to prevent complications, and daily wound care was performed to promote optimal healing. Psychological support was also provided to help her manage the emotional impact of the surgery and reconstruction, contributing to an overall improvement in her quality of life. The multidisciplinary approach, incorporating curative, reconstructive, and psychological care, was tailored to her needs as a seamstress, emphasizing the importance of a swift functional recovery. By one month post-surgery, she demonstrated improved mobility and psychological recovery. At the three-month follow-up, the patient showed no signs of recurrence or major complications, and her overall recovery had progressed well ( Figure 5).

## Discussion

Phyllodes tumors encompass a wide spectrum of fibroepithelial lesions, ranging from fibroadenomas to benign, borderline, and malignant phyllodes tumors, based on their histological characteristics. ^14, 15^ The term “phyllodes” is derived from the Latin word “phyllodium,” meaning “leaf-like,” referring to the tumor’s gross appearance, which is characterized by a large, lobulated, leaf-like structure. ^16, 17^

These tumors predominantly affect women aged 35 to 55 and are rare in adolescents or the elderly. ^18^ Although phyllodes tumors constitute only 0.3 to 0.5% of all breast neoplasms, with an incidence of approximately 2.1 cases per million women, they vary widely in behavior. ^19^ It is estimated that 85-90% of these tumors are benign, while 10-15% are malignant. ^20^ Giant phyllodes tumors, those exceeding 10 cm in diameter, account for around 20% of cases and carry a higher risk of malignancy.

Phyllodes tumors commonly arise in the right breast, and in approximately one-third of cases, they are multicentric. These tumors often present as rapidly enlarging, painless masses that distort the breast and stretch the skin, leading to potential complications such as pressure necrosis. The rapid growth and significant size are hallmark indicators of phyllodes tumors, distinguishing them from fibroadenomas, although their clinical presentations can be similar. This classical presentation was evident in the case we observed. ^21, 22^

For diagnosis, imaging techniques such as ultrasound, mammography, MRI, and core needle biopsy are employed. Among these, ultrasound and mammography are most frequently used as first-line diagnostic tools. ^20^ Ultrasound typically shows large, heterogeneous solid masses with lobulated margins and possible internal vascularity, while mammography reveals hyperdense masses, often occupying a significant portion of the breast. ^16, 23, 24^ Despite these findings, phyllodes tumors share imaging characteristics with fibroadenomas, making it challenging to distinguish between the two based on imaging alone. ^15^ Notably, phyllodes tumors do not exhibit pathognomonic features on either ultrasound or mammography. ^20^

Due to the size of these tumors, core needle biopsy or fine needle aspiration may not always provide a definitive diagnosis, as histological similarities between benign phyllodes tumors, fibroadenomas, and phyllodes sarcomas can complicate differentiation. ^24, 25^ Excisional biopsy, with careful assessment of surgical margins, is often necessary for accurate diagnosis. ^24^

Total mastectomy remains the cornerstone of treatment for phyllodes sarcoma, especially when they reach significant sizes and carry a high risk of recurrence. Experts recommend excising the tumor with at least a 1 cm margin, either during initial surgery or in cases of re-excision. Axillary lymph node dissection is not typically required for non-metastatic phyllode tumors, as lymph node involvement is rare. ^26, 27^

The psychological impact of mastectomy, especially following the removal of large tumors, is significant, as it can adversely affect body image and self-esteem. ^28, 29^ Immediate breast reconstruction using a latissimus dorsi myocutaneous flap offers a valuable option for restoring breast contour while addressing both functional and aesthetic needs. ^30^ This procedure involves the transfer of muscle, fat, and skin from the back, carefully preserving the vascular pedicle to ensure blood supply. The flap is then tunneled to the chest to reconstruct the breast, and it may be combined with implants or tissue expanders to optimize cosmetic results. Postoperative care focuses on maintaining flap viability, wound healing, and restoring shoulder function, with minimal long-term functional deficits. ^31, 32^

This approach effectively combines oncologic and aesthetic goals while significantly improving patients’ postoperative quality of life. Studies have highlighted the positive effects of breast reconstruction on quality of life, particularly regarding body image. This is especially true for younger women who value their physical appearance highly. Reconstruction not only helps restore physical form but also improves emotional well-being and self-esteem, facilitating a quicker return to normal activities through early mobilization and functional recovery. ^33– 36^

It is essential to integrate oncologic and reconstructive approaches to ensure comprehensive care. ^37^ While breast reconstruction restores physical form and emotional health, adjuvant therapies, such as radiotherapy, play a critical role in reducing local recurrence rates in malignant phyllodes tumors, though they do not impact overall survival or disease-free survival. ^15^ Chemotherapy is not routinely used, as its benefits are unproven, limiting its use to compassionate cases. Hormonal therapy is also not recommended, despite positive hormone receptor status in up to 75% of cases. In metastatic disease, treatment protocols for soft tissue sarcomas should be followed. ^15, 38^

## Conclusion

In conclusion, this case of a 40-year-old woman with a giant phyllodes sarcoma of the breast, successfully treated with total mastectomy and immediate latissimus dorsi flap reconstruction, illustrates the importance of a multidisciplinary approach in managing both the oncologic and aesthetic challenges posed by large tumors. This case emphasizes the need for timely surgical intervention, combined with effective reconstructive techniques, to minimize physical and psychological impacts, ensuring optimal recovery and quality of life for the patient. It also highlights the necessity of individualized treatment planning, particularly in cases where functional and aesthetic outcomes are critical.

## Consent

Written informed consent was obtained from the patient for the publication of their clinical details and/or images.

## Data Availability

No data are associated with this article.
