# Case Report: Giant Malignant Phyllodes Tumor of the Breast: Immediate Reconstruction with a Pedicled Latissimus Dorsi Myocutaneous Flap Following Total Mastectomy—A Case Report and Review of Reconstructive Considerations

**DOI:** 10.12688/f1000research.159059.2

**Published:** 2026-08-08

**Authors:** Dhekra Toumi, Chayma Cheikh Mohamed, Ahmed Hajji, Imen Ghaddab, Malak Medemagh, Hanane Lazreg, Yasmine Ben Ali, Sana Alouani, Jawaher Hammedi, Selma Souilah, Rihab Barouni, Hiba Ben Abdelhafidh, Sana bouakez, Thana Mahfoudhi, Olfa Zoukar, Haifa Bergaoui, Raja Faleh

**Affiliations:** 1 Obstetrics and Gynecology Department, Faculty of Medicine, Monastir Maternity and Neonatology Centre, University of Monastir, Monastir, Monastir, 5030, Tunisia

**Keywords:** Phyllodes sarcoma, Giant breast tumor, mastectomy, Latissimus dorsi flap reconstruction, Breast oncology, Reconstructive surgery, body image, quality of life

## Abstract

Phyllodes tumor of the breast is a rare fibroepithelial tumor, constituting less than 1% of all breast neoplasms. These tumors are characterized by rapid stromal growth and can reach significant sizes, particularly in malignant cases, necessitating aggressive surgical management due to their high recurrence risk. In this case report, we present a 40-year-old Tunisian woman diagnosed with a giant phyllodes sarcoma. The patient experienced considerable physical and psychological impacts, including impaired mobility, body image disruption, and professional limitations. Clinical examination and imaging confirmed the presence of a large lobulated mass in the left breast, with no signs of distant metastasis. A multidisciplinary team recommended a total mastectomy with immediate reconstruction using a latissimus dorsi myocutaneous flap. This approach aimed to restore breast contour while addressing both oncologic and aesthetic needs. The procedure was successful, with the excised tumor weighing 7 kg. Postoperatively, the patient received close monitoring and psychological support, emphasizing the importance of a holistic approach to recovery. This case underscores the complexities of managing giant phyllodes sarcoma, highlighting the value of a timely surgical intervention combined with effective reconstructive techniques. It also emphasizes the need for individualized treatment plans that consider both functional and aesthetic outcomes to optimize patient recovery and quality of life

## Introduction

Breast phyllodes tumors are rare fibroepithelial neoplasms accounting for less than 1% of all breast tumors. They are classified as benign, borderline, or malignant according to stromal cellularity, atypia, mitotic activity, stromal overgrowth, and tumor margins. Although malignant phyllodes tumors represent only a small proportion of all phyllodes tumors, they are characterized by rapid growth, aggressive local behavior, and a high risk of local recurrence, often requiring radical surgical treatment. ^1^ ^–^ ^4^

Giant malignant phyllodes tumors are exceptionally uncommon and constitute a major therapeutic challenge. Their rapid enlargement frequently results in breast deformity, skin ulceration, pain, functional impairment, and considerable psychological distress. From a surgical perspective, achieving complete oncologic resection while preserving acceptable aesthetic and functional outcomes is particularly challenging, especially when extensive soft-tissue defects remain following tumor excision. ^5^ ^–^ ^8^

Wide local excision with histologically negative margins remains the cornerstone of treatment. However, when breast-conserving surgery cannot ensure adequate oncologic margins, total mastectomy becomes necessary. In these situations, immediate breast reconstruction plays a fundamental role in restoring chest wall integrity, breast contour, body image, and overall quality of life. Among the available reconstructive techniques, the pedicled latissimus dorsi myocutaneous flap remains a reliable and versatile option because of its robust vascular supply, technical reproducibility, and ability to provide well-vascularized tissue for large chest wall defects. ^9^ ^–^ ^13^

Despite advances in breast reconstruction, reports describing immediate autologous reconstruction following resection of exceptionally large malignant phyllodes tumors remain scarce. To the best of our knowledge, the present case represents one of the largest malignant phyllodes tumors reported in the literature, with a surgical specimen weighing 7 kg. Beyond its exceptional size, this case highlights the reconstructive challenges associated with extensive oncologic defects and illustrates the role of immediate pedicled latissimus dorsi flap reconstruction in achieving satisfactory oncologic, functional, and aesthetic outcomes.

We therefore report the case of a 40-year-old woman with a giant malignant phyllodes tumor successfully treated by total mastectomy followed by immediate reconstruction using a pedicled latissimus dorsi myocutaneous flap. In addition to presenting the surgical management and postoperative outcome, we discuss the reconstructive rationale, recent advances in autologous breast reconstruction, and the current evidence regarding the oncologic safety of adjunctive fat grafting.

## Case presentation

This case report was prepared in accordance with the SCARE 2023 (Surgical CAse REport) Guidelines.

We report the case of a 40-year-old Tunisian woman, a seamstress by profession, with no family history of breast cancer and a past medical history significant only for thyroidectomy performed for a benign thyroid nodule.

The patient presented with a rapidly enlarging left breast mass that had progressively increased in size over five months, ultimately causing marked breast asymmetry and severe impairment of her quality of life.

Beyond its exceptional size, the tumor produced significant functional limitations. The patient reported persistent chest heaviness, chronic thoracic pain, difficulty maintaining an upright posture, impaired upper limb movements, disturbed sleep, and considerable limitations in performing routine daily activities such as bending, walking, dressing, and prolonged sitting. These symptoms had also become professionally disabling, preventing her from carrying out her work as a seamstress and resulting in a substantial reduction in income.

The psychological burden was equally profound. Progressive breast deformity led to severe body image disturbance, social withdrawal, loss of self-confidence, and persistent anxiety regarding the possibility of malignancy.

**Figure 1.**
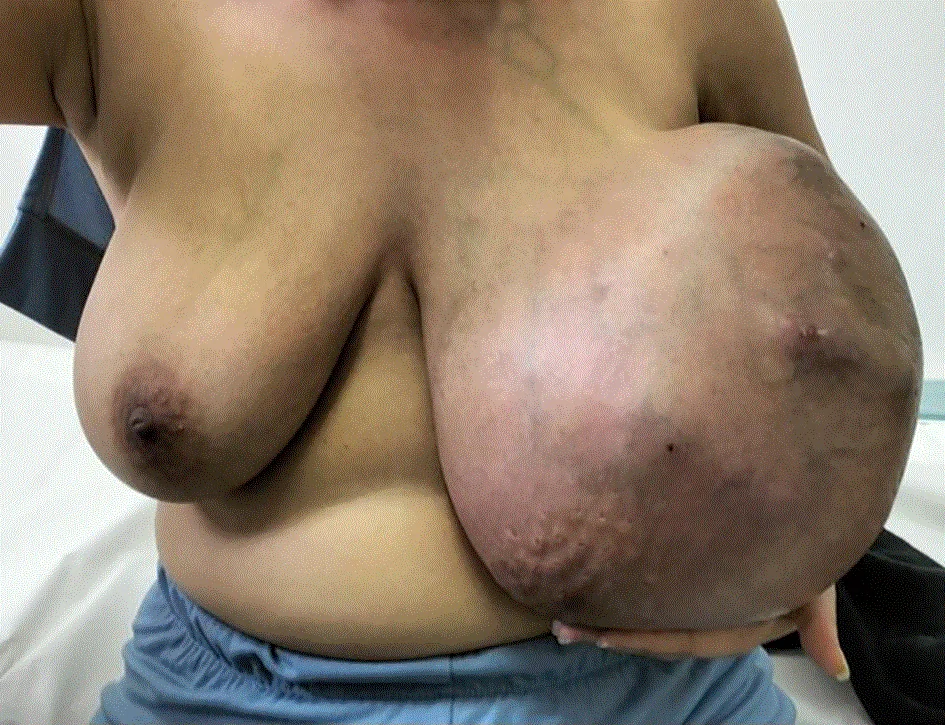
Giant Phyllodes Tumor on Clinical Examination: A 40-year-old female patient presenting presents with a giant phyllodes tumor (30 × 40 cm) in the left breast, accompanied by dilated subcutaneous vessels.

Clinical examination revealed a well-circumscribed, firm, mobile, lobulated mass occupying the entire left breast, measuring approximately 30 × 40 cm ( Figure 1).

**Figure 2.**
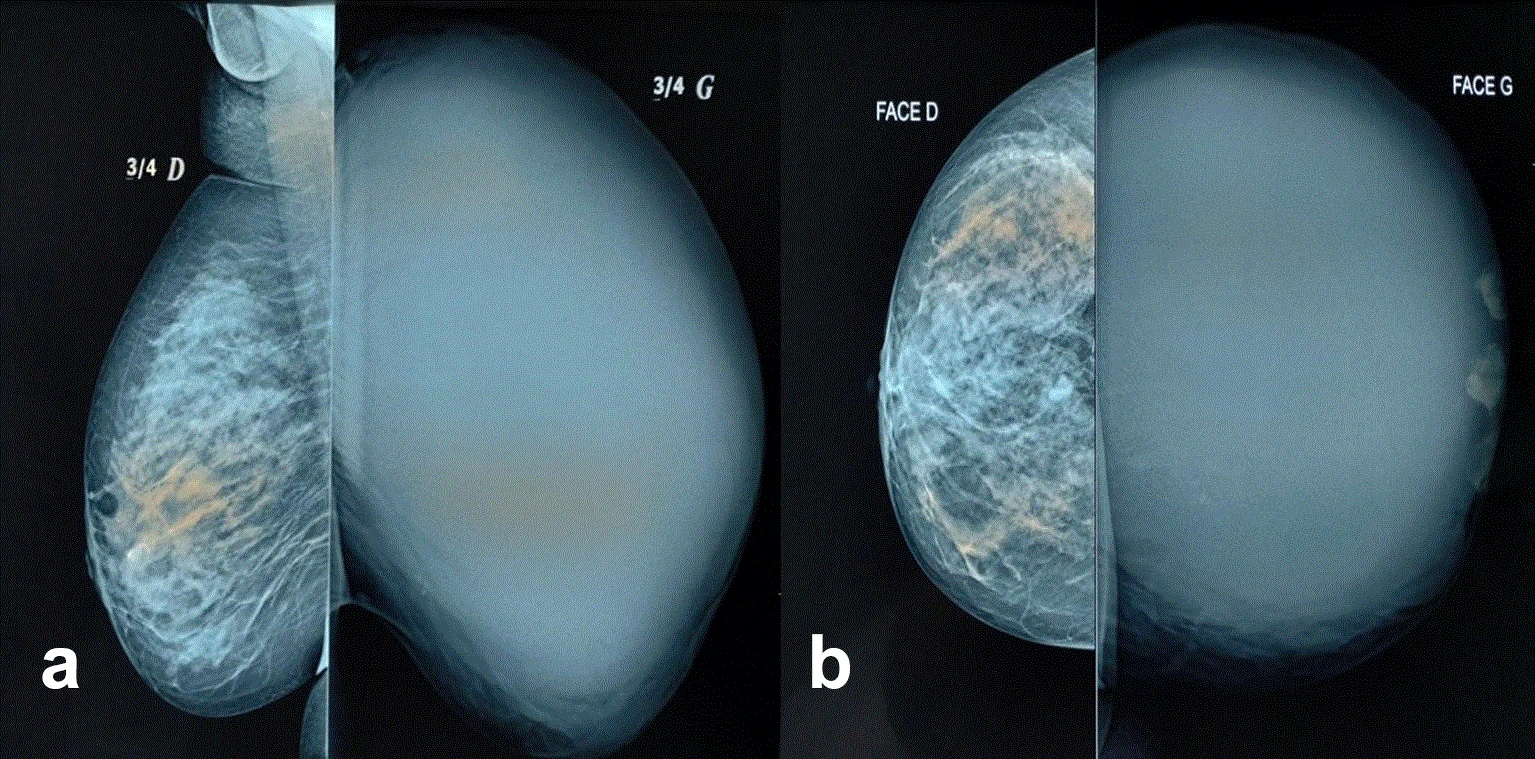
Initial mammogram: (a) Oblique view, (b) Frontal view.

The overlying skin was markedly stretched and erythematous, with prominent dilated superficial veins, focal ulcerations, and peau d'orange appearance secondary to skin tension. No palpable axillary lymphadenopathy was identified, and no clinical signs of distant metastatic disease were observed.

Initial breast ultrasonography suggested proliferative mastitis complicated by superinfection, whereas bilateral mammography demonstrated a giant lobulated mass measuring approximately 40 × 30 cm, associated with marked architectural distortion of the left breast ( Figure 2).

Contrast-enhanced thoraco-abdominopelvic computed tomography confirmed a large breast mass associated with ipsilateral axillary lymphadenopathy but revealed no evidence of distant metastatic disease. Ultrasound-guided core needle biopsy established the diagnosis of high-grade malignant phyllodes tumor (phyllodes sarcoma).

Following discussion at a multidisciplinary tumor board, including breast surgeons, reconstructive surgeons, radiologists, pathologists, and oncologists, the indication for total mastectomy with a minimum 1-cm oncologic margin followed by immediate autologous breast reconstruction was unanimously retained.

The principal reconstructive challenge resulted from the anticipated extensive chest wall soft-tissue defect following complete oncologic excision. After careful preoperative assessment of the anticipated tissue loss and donor-site availability, a pedicled latissimus dorsi myocutaneous flap was selected because of its reliable vascular anatomy, versatility, and ability to provide well-vascularized tissue for immediate coverage of the expected defect.

**Figure 3.**
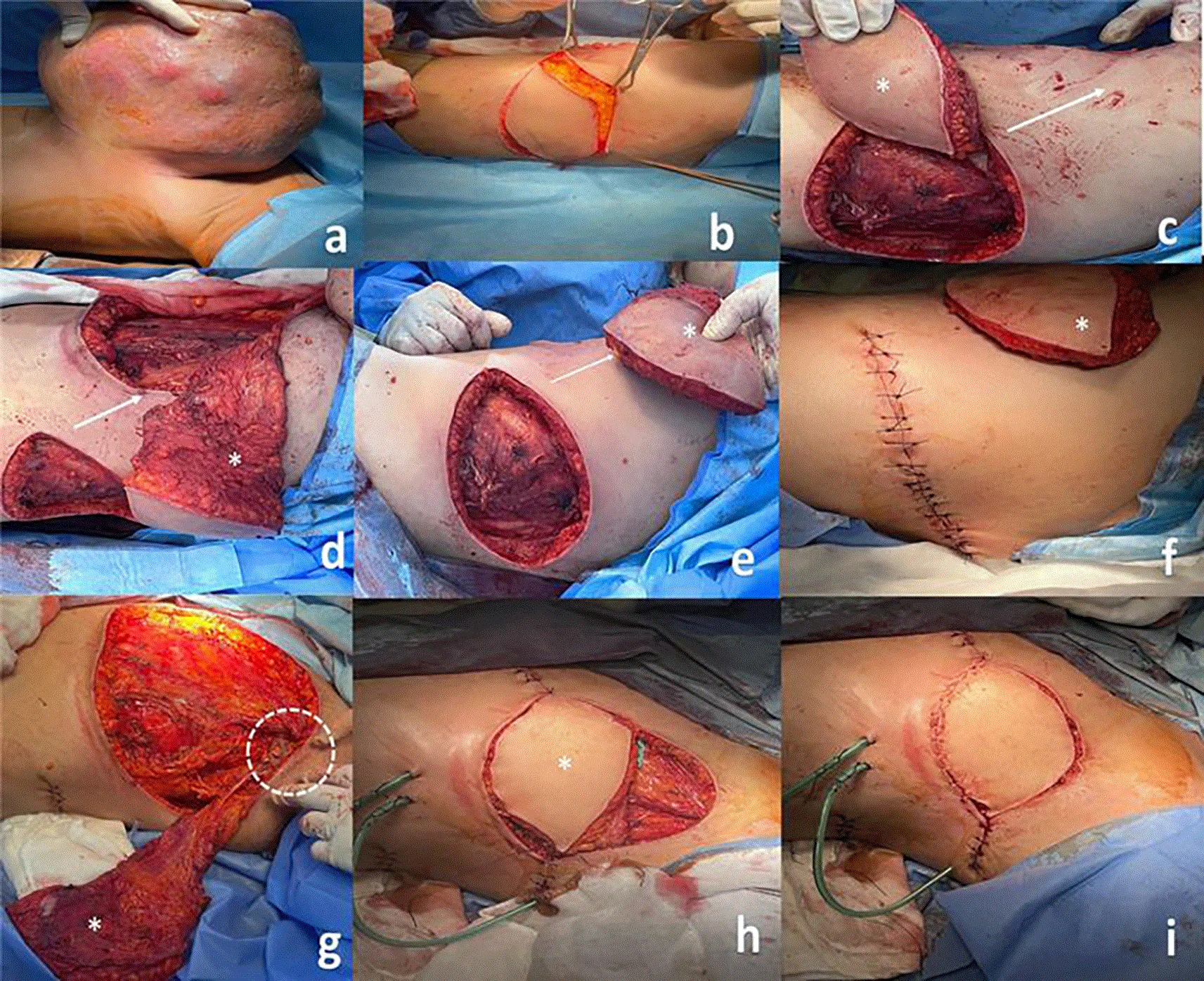
Comprehensive procedure for latissimus dorsi myocutaneous flap harvest and transfer for breast reconstruction. (a) Comprehensive preoperative evaluation (b) Dorsal incision made to harvest the latissimus dorsi myocutaneous flap (c) Creation of a subcutaneous tunnel from the dorsal region to the chest to allow flap transfer: Careful dissection and tissue detachment to create a subcutaneous pocket, while ensuring the preservation of the flap's vascular supply. The white arrow in the image indicates the direction of the tissue dissection and the subcutaneous tunnel created to allow for the flap's passage. (d), (e) Transfer of the flap through the subcutaneous tunnel to the mastectomy bed. (f ) The donor site incision is then meticulously sutured. (g) Careful preservation of key blood vessels during flap harvest, followed by precise flap positioning on the mastectomy bed. The dashed circle in the figure indicates the preserved vascularization, while the asterisk marks the flap. (h) Shaping of the flap to restore breast volume and contour. (i) Attachment of the flap to ensure proper reconstruction.

Under general anesthesia, a total mastectomy was first performed while respecting oncologic principles and achieving macroscopically negative surgical margins. Reconstruction was subsequently undertaken through harvest of a pedicled latissimus dorsi myocutaneous flap incorporating a 10 × 15 cm skin paddle ( Figure 3).

The thoracodorsal vascular pedicle was meticulously preserved throughout flap elevation to ensure optimal perfusion. The flap was transferred through a carefully created subcutaneous tunnel into the mastectomy defect, where it was shaped to restore breast contour while providing complete coverage of the extensive soft-tissue defect. The donor site was closed primarily without excessive tension.

Immediate contralateral balancing surgery was intentionally not performed. Given the exceptionally large tumor burden, the primary objective was complete oncologic resection followed by reliable reconstruction of the extensive post-mastectomy defect. Furthermore, because the patient presented with a high-grade malignant phyllodes tumor requiring consideration of adjuvant treatment, secondary aesthetic procedures were deliberately deferred until completion of oncologic management, should they become clinically indicated.

**Figure 4.**
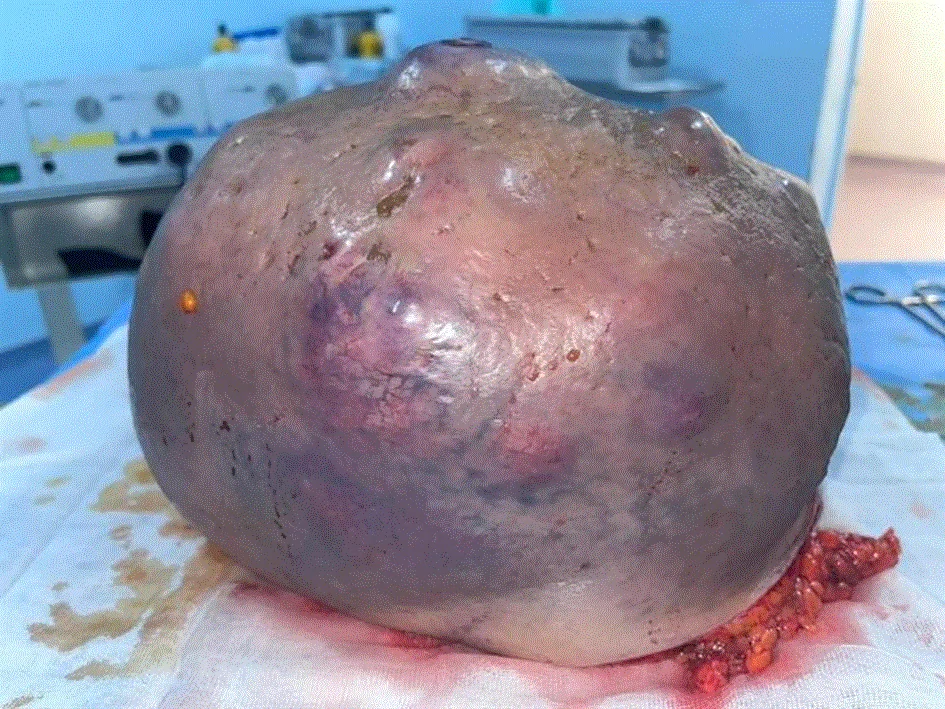
Surgical specimen, tumor weighing 7 kg.

The surgical specimen weighed 7 kg ( Figure 4).

Histopathological examination confirmed the diagnosis of a high-grade malignant phyllodes tumor, with tumor-free surgical margins.

Postoperatively, the patient received routine flap monitoring, daily wound care, analgesia, physiotherapy, and psychological support. Particular attention was paid to flap perfusion, wound healing, shoulder mobility, and functional recovery.

The postoperative course was uneventful. No flap-related complications, wound dehiscence, infection, hematoma, seroma requiring intervention, or skin necrosis occurred. Progressive functional recovery was observed during follow-up, with marked improvement in posture, shoulder mobility, and overall quality of life.

**Figure 5.**
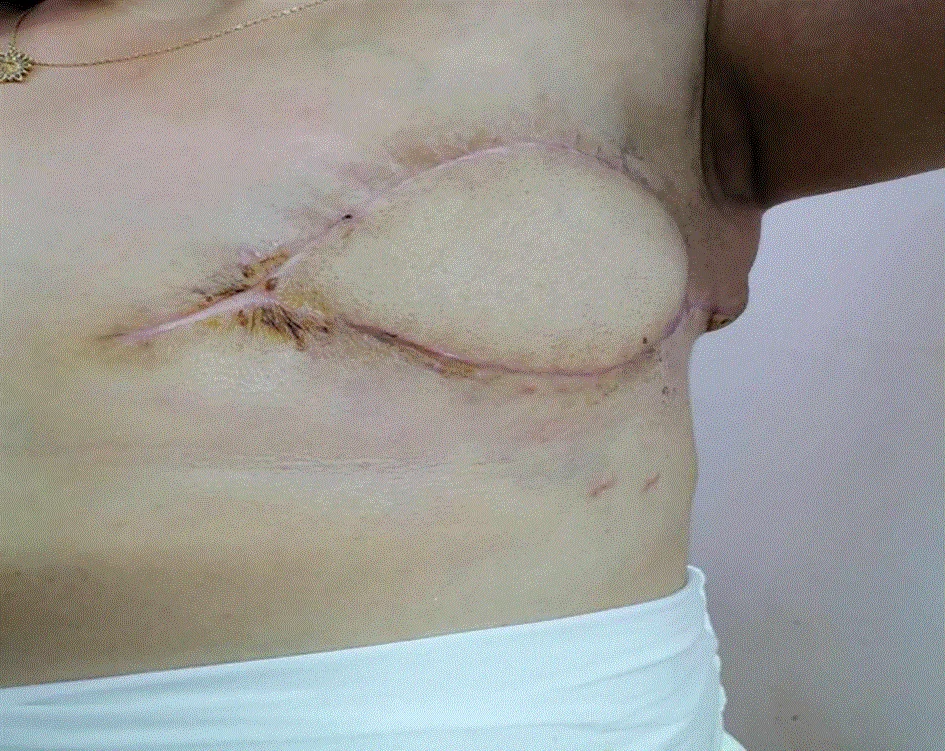
Appearance of breast reconstruction site three months post-surgery.

At three months, clinical examination demonstrated complete wound healing, excellent flap viability, satisfactory breast contour, and no evidence of local recurrence or major postoperative complications ( Figure 5). The patient reported substantial improvement in both physical function and psychological well-being and expressed satisfaction with the reconstructive outcome.

## Discussion

Phyllodes tumors are rare fibroepithelial breast neoplasms that encompass a wide histological spectrum ranging from benign to borderline and malignant lesions. Malignant phyllodes tumors account for only 10–15% of all phyllodes tumors but are characterized by rapid growth, aggressive local behavior, and a high risk of local recurrence, making complete surgical excision with negative margins the cornerstone of treatment. ^14^ ^,^ ^15^ Although giant phyllodes tumors are uncommon, they represent a major surgical challenge because of their massive size, the extensive soft-tissue defects created after resection, and the complex reconstructive considerations required to restore both function and body image.

Imaging modalities, including ultrasound, mammography, magnetic resonance imaging, and core needle biopsy, remain essential components of the diagnostic work-up. However, differentiating malignant phyllodes tumors from large fibroadenomas remains difficult because no imaging feature is pathognomonic. Consequently, histopathological examination following surgical excision remains the diagnostic gold standard. ^15^ ^,^ ^20^ ^,^ ^23^ ^–^ ^25^

Wide surgical excision with histologically negative margins remains the recommended treatment for malignant phyllodes tumors. Current international guidelines recommend margins of at least 1 cm whenever feasible to minimize local recurrence. ^26^ ^,^ ^27^ In patients presenting with exceptionally large tumors, breast-conserving surgery is generally impossible because adequate oncologic margins would result in unacceptable cosmetic deformity. Therefore, total mastectomy becomes the most appropriate surgical option.

Breast reconstruction following mastectomy for giant phyllodes tumors presents unique reconstructive challenges. Unlike conventional mastectomy performed for breast carcinoma, excision of giant tumors frequently results in extensive chest wall soft-tissue defects requiring reliable vascularized tissue coverage. In our patient, the tumor measured 30 × 40 cm and weighed 7 kg, requiring a wide oncologic resection that created a substantial loss of soft tissue. For this reason, immediate reconstruction using a pedicled latissimus dorsi myocutaneous flap was selected. The harvested skin paddle measured **10 × 15 cm**, providing sufficient well-vascularized tissue to achieve durable coverage of the chest wall defect while restoring breast contour. The latissimus dorsi flap remains one of the most versatile reconstructive options in this setting because of its reliable vascular anatomy, technical reproducibility, relatively low donor-site morbidity, and ability to tolerate potential adjuvant radiotherapy. ^30^ ^–^ ^32^

Contralateral balancing surgery was deliberately not performed during the initial procedure. Although contralateral symmetrization can improve aesthetic outcomes in selected patients, our priority was complete oncologic resection followed by reliable reconstruction of the extensive chest wall defect. Furthermore, the patient had a high-grade malignant phyllodes sarcoma with an anticipated need for adjuvant treatment and an initially unfavorable oncologic prognosis. Consequently, aesthetic refinement was intentionally deferred until completion of oncologic management. Should long-term disease control be achieved, delayed contralateral symmetrization remains a reasonable secondary option according to the patient’s expectations and clinical evolution.

Recent advances in autologous breast reconstruction have further expanded the role of the latissimus dorsi flap through the development of the **fat-augmented latissimus dorsi (FALD) flap**. This technique combines the structural support provided by the transferred muscle with autologous fat grafting, allowing greater breast volume while avoiding prosthetic implants. Santanelli di Pompeo et al. reported excellent aesthetic outcomes and a high safety profile using this approach, highlighting its usefulness particularly in patients requiring moderate-to-large breast reconstruction. ^39^ In addition, staged autologous fat grafting performed after initial reconstruction may further improve breast contour and volume by taking advantage of the vascularized scaffold created by the latissimus dorsi flap. Although additional fat grafting was not required in our patient because satisfactory breast volume and contour were achieved after immediate reconstruction, FALD represents an attractive option for secondary refinement in appropriately selected patients.

Historically, concerns have been raised regarding the oncologic safety of autologous fat grafting following breast cancer surgery because of the theoretical interaction between adipose-derived stem cells and residual tumor cells. However, accumulating clinical evidence has largely alleviated these concerns. In a matched case-control study including approximately **1000 postmastectomy breast reconstructions**, Sorotos et al. demonstrated that autologous fat transfer was **not associated with increased local recurrence, regional recurrence, distant metastasis, or reduced overall survival**, supporting its oncologic safety when performed in appropriately selected patients. ^40^ These findings further support the growing role of lipofilling as a valuable adjunct to autologous breast reconstruction.

The exceptional size of the present tumor deserves particular emphasis. Giant phyllodes tumors are generally defined as tumors larger than 10 cm or weighing more than 1 kg. Although several isolated cases have been reported, tumors exceeding 5 kg remain exceedingly rare. To the best of our knowledge, the present specimen weighed **7 kg**, making it **the second heaviest malignant phyllodes tumor reported in the literature**. Only the case described by **Dong Xia et al.**, with an ex vivo specimen weighing **9.79 kg**, exceeded the weight observed in our patient. ^41^ In comparison, the case reported by **Albalawi et al.** weighed **5.4 kg**, which remains substantially smaller than the present case. ^42^ Beyond its exceptional weight, our case illustrates the considerable reconstructive challenge associated with immediate breast reconstruction following resection of extremely large malignant phyllodes tumors and highlights the feasibility of achieving satisfactory oncologic and aesthetic outcomes using a pedicled latissimus dorsi flap.

Finally, this case underscores the importance of a multidisciplinary approach integrating breast surgeons, reconstructive surgeons, pathologists, radiologists, oncologists, and psychological support. Beyond achieving complete oncologic resection, successful management of giant phyllodes tumors should also address postoperative function, body image, quality of life, and long-term aesthetic rehabilitation through individualized reconstructive strategies tailored to each patient’s clinical condition and oncologic prognosis.

## Conclusion

This case report describes the successful management of one of the largest malignant phyllodes tumors reported in the literature, weighing 7 kg, through total mastectomy followed by immediate reconstruction using a pedicled latissimus dorsi myocutaneous flap. It highlights that extensive oncologic resection of giant phyllodes tumors can be safely combined with immediate autologous reconstruction to restore chest wall coverage, breast contour, and postoperative quality of life.

Beyond its exceptional size, this case emphasizes the importance of individualized multidisciplinary decision-making that integrates oncologic safety, reconstructive planning, functional recovery, and psychological rehabilitation. The pedicled latissimus dorsi flap proved to be a reliable and versatile reconstructive option for managing the extensive soft-tissue defect created after radical tumor excision.

Given the rarity of giant malignant phyllodes tumors, additional well-documented case reports and larger clinical series are needed to further define optimal reconstructive strategies and improve the evidence base for the surgical management of these exceptionally challenging tumors.

## Consent

Written informed consent was obtained from the patient for the publication of their clinical details and/or images.

## Data Availability

No data are associated with this article.
