# Supplementary material for: Case Report: Giant Malignant Phyllodes Tumor of the Breast: Immediate Reconstruction with a Pedicled Latissimus Dorsi Myocutaneous Flap Following Total Mastectomy—A Case Report and Review of Reconstructive Considerations
Source: F1000Res. 2026 Aug 8;14:130. Originally published 2025 Jan 27. [Version 2] doi: 10.12688/f1000research.159059.2 (PMC13563010; doi:10.12688/f1000research.159059.2)
Supplement: Peer review history

# Reviewer report: Reviewer response for version 2

**DOI:** 10.5256/f1000research.207721.r515771

**Published:** 2026-08-31

**Authors:** Nesrine SOUAYEH

**Affiliations:** 1 Ben Arous Régional Hospital, Obstetrics and Gynaecology Department. Faculty of Medicine, University of Tunis el Manar, Tunis, Tunisia

This case report describes the management of a 40-year-old woman with an exceptionally large high-grade malignant phyllodes tumor of the breast. The tumor developed rapidly over five months, reached approximately 30 × 40 cm, and caused major physical, functional, professional, and psychological impairment. Imaging and core needle biopsy established the diagnosis, with CT showing no distant metastatic disease. Following multidisciplinary discussion, total mastectomy with adequate oncological margins and immediate reconstruction using a pedicled latissimus dorsi myocutaneous flap was performed. The surgical specimen weighed 7 kg. Histopathology confirmed a high-grade malignant phyllodes tumor with tumor-free margins. The postoperative course was uncomplicated, with good flap viability, satisfactory breast contour, and improvement in shoulder mobility, function and quality of life at three months.

 The revised manuscript is substantially improved and adequately addresses the points raised during the previous review, including adherence to the SCARE 2023 guidelines, additional operative details, the 10 × 15 cm skin paddle dimensions, the rationale for the choice of latissimus dorsi reconstruction, and the decision to defer contralateral symmetrization. The discussion has also been expanded to address fat-augmented latissimus dorsi reconstruction, autologous fat grafting and the exceptional size of the tumor in comparison with previously reported cases.

 F1000Research assessment questions

**1. Is the background of the case’s history and progression described in sufficient detail? — Yes.**

 The patient's clinical history, rapid tumor progression, symptoms, functional and psychological consequences, physical examination findings, diagnostic work-up and multidisciplinary management are clearly and sufficiently described.

**2. Are enough details provided of the physical examination, diagnostic tests, treatment given and outcomes? — Yes.**

 The manuscript provides adequate clinical and imaging findings, pathological diagnosis, surgical details and postoperative outcomes. The description of the latissimus dorsi flap, including its dimensions and surgical rationale, makes the reconstructive approach sufficiently understandable and reproducible.

**3. Is sufficient discussion included of the importance of the findings and their relevance to future understanding of disease processes, diagnosis or treatment? — Yes.**

 The discussion appropriately emphasizes the rarity and aggressive nature of malignant phyllodes tumors, the importance of complete surgical excision, and the reconstructive challenges associated with giant tumors. It also places the case in the context of contemporary reconstructive options and compares the tumor size with previously published giant phyllodes tumors.

**4. Is the case presented with sufficient detail to be useful for other practitioners? — Yes.**

 The case provides valuable practical information for clinicians involved in breast oncology and reconstructive surgery. In particular, it illustrates the importance of multidisciplinary decision-making and demonstrates the feasibility of immediate autologous reconstruction following extensive resection of a giant malignant phyllodes tumor.

 I found the revised manuscript clinically interesting, relevant and scientifically sound. The authors have satisfactorily addressed the issues raised during the previous review and have strengthened both the clinical presentation and the discussion.

**No mandatory revisions are required for scientific soundness. I recommend approval of the manuscript.**

Are enough details provided of any physical examination and diagnostic tests, treatment given and outcomes?

Yes

Is the case presented with sufficient detail to be useful for other practitioners?

Yes

Is sufficient discussion included of the importance of the findings and their relevance to future understanding of disease processes, diagnosis or treatment?

Yes

Is the background of the case’s history and progression described in sufficient detail?

Yes

Reviewer Expertise:

Gynaecology, breast cancer,

I confirm that I have read this submission and believe that I have an appropriate level of expertise to confirm that it is of an acceptable scientific standard.

# Reviewer report: Reviewer response for version 2

**DOI:** 10.5256/f1000research.207721.r509141

**Published:** 2026-08-19

**Authors:** Guido Firmani

**Affiliations:** 1 Sapienza University Rome, Rome, Italy

I commend the authors for their comprehensive revisions. Well done.

Are enough details provided of any physical examination and diagnostic tests, treatment given and outcomes?

Partly

Is the case presented with sufficient detail to be useful for other practitioners?

Yes

Is sufficient discussion included of the importance of the findings and their relevance to future understanding of disease processes, diagnosis or treatment?

Partly

Is the background of the case’s history and progression described in sufficient detail?

Yes

Reviewer Expertise:

Plastic surgery, breast reconstruction.

I confirm that I have read this submission and believe that I have an appropriate level of expertise to confirm that it is of an acceptable scientific standard.

# Reviewer report: Reviewer response for version 1

**DOI:** 10.5256/f1000research.174733.r370937

**Published:** 2025-03-24

**Authors:** Guido Firmani

**Affiliations:** 1 Sapienza University Rome, Rome, Italy

I commend the authors for their case report. This report is compelling, and the figures are convincing. I believe this is deserving of interest to the readership. However, I would appreciate if you could provide some additional information. Please see my comments listed below:

- Please make sure that your report abides by the SCARE checklist for case reports, which helps ensure that appropriate standards have been followed when preparing your manuscript. If this has already been done, then please acknowledge this in your manuscript.
- I would appreciate some added surgical details. It appears that you performed an autologous LD flap. What were the dimensions of the skin paddle you harvested? To account to differences in size between the affected side and the contralateral breast, have you considered performing a balancing procedure?
- Did you consider augmenting your LD volume over time with added surgical procedures? It could be viable to perform a FALD with subsequent autologous fat grafts to achieve a larger volume using the scaffolding created by the LD muscle and overlying skin paddle. [PMID: **35907690]**
- Do you have concerns of oncologic safety regarding the utility of autologous fat grafting in breast cancer patients? Please acknowledge evidence from a case-control study on 1000 breast cancer patients which found it not to increase local recurrence rates. [PMID: 35943964]
- Phyllodes tumors are considered “giant” when weighing over 1 kg. Your case well exceeds that threshold and is the second heaviest report in literature, after the one reported by Dong Xia et al. [2010 (Ref-3)] (doi: 10.1007/s10330-010-0688-0) weighing 9.79 kg ex vivo! The third heaviest report was 5.4 kg. [PMID: 30034663]. Please consider addressing these reports from literature.

Are enough details provided of any physical examination and diagnostic tests, treatment given and outcomes?

Partly

Is the case presented with sufficient detail to be useful for other practitioners?

Yes

Is sufficient discussion included of the importance of the findings and their relevance to future understanding of disease processes, diagnosis or treatment?

Partly

Is the background of the case’s history and progression described in sufficient detail?

Yes

Reviewer Expertise:

Plastic surgery, breast reconstruction.

I confirm that I have read this submission and believe that I have an appropriate level of expertise to confirm that it is of an acceptable scientific standard, however I have significant reservations, as outlined above.

## References

[1] : Total breast reconstruction with the fat-augmented latissimus dorsi (FALD) flap: High safety in a single-center uncontrolled case series. *J Plast Reconstr Aesthet Surg* .2022;75(9) : 10.1016/j.bjps.2022.06.0523004-3013 10.1016/j.bjps.2022.06.052 35907690

[2] : Oncologic Outcome of 1000 Postmastectomy Breast Reconstructions with Fat Transfer: A Single-Center, Matched Case-Control Study. *Plast Reconstr Surg* .2022;150: 10.1097/PRS.00000000000094944S-12S 10.1097/PRS.0000000000009494 35943964

[3] : Giant phyllodes tumor of the breast: a case report. *The Chinese-German Journal of Clinical Oncology* .2010;9(11) : 10.1007/s10330-010-0688-0674-676 10.1007/s10330-010-0688-0

[4] : A huge phyllodes tumor in the breast: a case report. *Electron Physician* .2018;10(6) : 10.19082/69516951-6955 10.19082/6951 30034663 PMC6049979

# Response

**Published:** 2026-07-30

**Authors:** chayma cheikhmohamed

We sincerely thank the reviewer for the thorough evaluation of our manuscript and for the constructive and insightful comments. We are grateful for the positive assessment of our work and appreciate the valuable suggestions, which have significantly improved the quality and scientific rigor of our manuscript. We have carefully revised the manuscript accordingly, and all comments have been addressed as detailed below.

**Comment 1**

 Please make sure that your report abides by the SCARE checklist for case reports, which helps ensure that appropriate standards have been followed when preparing your manuscript. If this has already been done, then please acknowledge this in your manuscript.

**Response**

 Thank you for this valuable suggestion.

 The manuscript has now been revised in accordance with the **SCARE 2023 Guidelines for Surgical CAse REports**. We have explicitly stated in the *Case Presentation* section that the manuscript was prepared following the SCARE recommendations. In addition, the completed **SCARE 2023 checklist** has been included as supplementary material in accordance with the journal recommendations.

**Comment 2**

 I would appreciate some added surgical details. It appears that you performed an autologous LD flap. What were the dimensions of the skin paddle you harvested? To account to differences in size between the affected side and the contralateral breast, have you considered performing a balancing procedure?

**Response**

 We sincerely thank the reviewer for this important observation.

 Additional operative details have now been incorporated into the *Case Presentation* section to provide a more comprehensive description of the reconstructive procedure.

 Specifically, we have added that the **latissimus dorsi myocutaneous flap was harvested with a 10 × 15 cm skin paddle**, designed according to the anticipated post-mastectomy defect while allowing primary closure of the donor site.

 We have also clarified the rationale for selecting a pedicled latissimus dorsi flap. Because the patient presented with an exceptionally large malignant phyllodes tumor, wide oncologic resection resulted in a substantial chest wall soft-tissue defect. Therefore, immediate reconstruction using a well-vascularized myocutaneous flap was considered the most reliable option to achieve durable coverage while restoring breast contour.

 Regarding contralateral balancing surgery, this option was discussed during reconstructive planning. However, because the patient had a **high-grade malignant phyllodes sarcoma**, with an anticipated need for adjuvant treatment and an initially unfavorable oncologic prognosis, the surgical priority was complete oncologic resection and reliable reconstruction rather than immediate aesthetic symmetry. Consequently, contralateral symmetrization was intentionally deferred. We have now clarified this point in both the *Case Presentation* and the *Discussion*. Delayed balancing surgery remains a possible secondary option depending on the patient's long-term oncologic outcome and aesthetic expectations.

**Comment 3**

 Did you consider augmenting your LD volume over time with added surgical procedures? It could be viable to perform a FALD with subsequent autologous fat grafts to achieve a larger volume using the scaffolding created by the LD muscle and overlying skin paddle.

**Response**

 We thank the reviewer for this excellent and highly relevant suggestion.

 We fully agree that **fat-augmented latissimus dorsi (FALD) reconstruction** has become an important advancement in autologous breast reconstruction.

 Following the reviewer's recommendation, we have expanded the Discussion to include the recent work by **Santanelli di Pompeo et al.**, describing the FALD technique and its favorable aesthetic outcomes and safety profile.

 In the present case, the conventional latissimus dorsi flap provided sufficient soft-tissue volume to achieve satisfactory breast contour immediately after resection of the giant tumor. Therefore, additional volume augmentation with staged autologous fat grafting was not considered necessary during the initial reconstructive phase.

 Nevertheless, we now discuss FALD as an attractive secondary reconstructive option for patients requiring additional breast volume or contour refinement after completion of primary treatment.

**Comment 4**

 Do you have concerns of oncologic safety regarding the utility of autologous fat grafting in breast cancer patients? Please acknowledge evidence from a case-control study on 1000 breast cancer patients which found it not to increase local recurrence rates.

**Response**

 We appreciate this important comment.

 The Discussion has been expanded to address the current evidence regarding the oncologic safety of autologous fat grafting.

 We have incorporated the study by **Sorotos et al.**, which evaluated approximately **1000 postmastectomy breast reconstructions** using autologous fat transfer. This matched case-control study demonstrated that lipofilling was **not associated with increased local recurrence, regional recurrence, distant metastasis, or decreased overall survival**, supporting its oncologic safety when performed in appropriately selected patients.

 These findings have now been included in the revised Discussion to provide a more balanced and up-to-date overview of contemporary reconstructive strategies.

**Comment 5**

 Phyllodes tumors are considered "giant" when weighing over 1 kg. Your case well exceeds that threshold and is the second heaviest report in literature, after the one reported by Dong Xia et al. weighing 9.79 kg ex vivo. The third heaviest report was 5.4 kg. Please consider addressing these reports from literature.

**Response**

 We sincerely thank the reviewer for highlighting these important reports.

 Following this recommendation, we performed an updated literature review and substantially expanded the Discussion.

 We now emphasize that giant phyllodes tumors are generally defined as tumors larger than 10 cm or weighing more than 1 kg. We further highlight the exceptional nature of our case by comparing it with previously published reports.

 To the best of our knowledge, the present specimen weighed **7 kg**, making it **the second heaviest malignant phyllodes tumor reported in the literature**. Only the case reported by **Xia et al.**, with an ex vivo specimen weighing **9.79 kg**, exceeds the present case, whereas the tumor described by **Albalawi et al.** weighed **5.4 kg**.

 We believe that this comparison better emphasizes the rarity of our case and reinforces its scientific interest for surgeons involved in breast oncology and reconstructive surgery.

**Comment 6**

 Are enough details provided of any physical examination and diagnostic tests, treatment given and outcomes?

**Response**

 We appreciate the reviewer's observation.

 The manuscript has been revised to provide a more detailed description of the surgical management and reconstructive procedure.

 Specifically, additional information regarding flap design, flap dimensions, reconstructive rationale, postoperative management, and the decision-making process regarding contralateral symmetrization has been incorporated.

 These additions improve the reproducibility of the surgical technique and provide greater practical value for readers.

**Comment 7**

 Is sufficient discussion included of the importance of the findings and their relevance to future understanding of disease processes, diagnosis or treatment?

**Response**

 Thank you for this valuable suggestion.

 The Discussion has been substantially revised and expanded.

 In particular, we have:

- provided a more detailed justification for choosing immediate reconstruction with a pedicled latissimus dorsi flap after extensive oncologic resection;
- discussed the rationale for postponing contralateral balancing surgery in the context of a high-grade malignant tumor requiring adjuvant treatment;
- incorporated recent evidence regarding **fat-augmented latissimus dorsi (FALD) reconstruction** and staged autologous fat grafting;
- discussed the current evidence supporting the **oncologic safety of autologous fat transfer**, based on the largest available matched case-control study;
- highlighted the exceptional nature of our case by comparing it with the largest giant phyllodes tumors reported in the literature.

We believe these revisions significantly strengthen the scientific discussion and better position our case within the current literature on giant malignant phyllodes tumors and breast reconstruction.

 Once again, we sincerely thank the reviewer for the thoughtful and constructive comments, which have considerably improved the quality of our manuscript. We greatly appreciate the reviewer's careful assessment and valuable recommendations. The manuscript has been thoroughly revised to address each point raised, including additional surgical details, an expanded discussion supported by recent literature, and a clearer presentation of the originality and clinical significance of this exceptionally rare case. We believe these revisions have strengthened the scientific rigor, clinical relevance, and educational value of our work. We hope that the revised version satisfactorily addresses all of the reviewer's concerns and will now be considered suitable for publication in *F1000Research*.
